# Supplementary material for: Progeny-based genomic selection reveals untapped genetic potential in an underutilized medicinal plant, Perilla frutescens
Source: Mol Breed. 2026 Aug 3;46(8):77. doi: 10.1007/s11032-026-01700-6 (PMC13433675; doi:10.1007/s11032-026-01700-6)
Supplement: Supplementary file 1 [file 11032_2026_1700_MOESM1_ESM.docx]

**TableS1. Individual IDs of the eight crosses used for generating the G₂ generation.**

|  | **Individual 1** | **Individual 2** |
| --- | --- | --- |
| **Crs1** | S827F5_084_2 | S827F5_106_3 |
| **Crs2** | S840F5_004_2 | S827F5_084_2 |
| **Crs3** | S840F5_038_1 | S827F5_084_2 |
| **Crs4** | S840F5_004_2 | S827F5_106_3 |
| **Crs5** | S840F5_038_1 | S827F5_106_3 |
| **Crs6** | S840F5_004_2 | S840F5_038_1 |
| **Crs7** | S827F5_009_3 | S827F5_084_2 |
| **Crs8** | S827F5_163_1 | S844F5_065_2 |

**TableS2. Phenotypic value of G_2_ generation for each cross and ‘Sekiho’.**

|  | **Perillaldehyde** | | | **Rosmarinic Acid** | | | **Anthocyanin** | | | |
| --- | --- | --- | --- | --- | --- | --- | --- | --- | --- | --- |
|  | **Mean** | **Max** | **Variance** | **Mean** | **Max** | **Variance** | **Mean** | **Max** | **Variance** |  |
| **Crs1** | 6.64 | 10.36 | 2.26 | 24.63 | 36.56 | 18.62 | 2.79 | 3.72 | 0.17 |  |
| **Crs2** | 4.87 | 10.28 | 5.60 | 25.56 | 35.13 | 20.40 | 1.39 | 3.57 | 0.57 |  |
| **Crs3** | 4.62 | 9.47 | 6.12 | 23.90 | 36.95 | 27.16 | 2.80 | 3.81 | 0.19 |  |
| **Crs4** | 3.08 | 9.58 | 3.53 | 25.88 | 37.54 | 19.83 | 1.41 | 3.30 | 0.71 |  |
| **Crs5** | 0.48 | 1.39 | 0.10 | 31.22 | 42.49 | 19.60 | 2.16 | 3.12 | 0.09 |  |
| **Crs6** | 4.14 | 9.21 | 5.70 | 27.27 | 43.91 | 38.50 | 2.49 | 3.36 | 0.07 |  |
| **Crs7** | 5.50 | 11.37 | 3.55 | 20.33 | 38.21 | 33.02 | 2.42 | 3.51 | 0.13 |  |
| **Crs8** | 3.53 | 5.72 | 0.62 | 21.38 | 30.92 | 12.97 | 3.57 | 4.50 | 0.10 |  |
| **‘Sekiho’** | 4.56 | | | 19.43 | | | 3.60 | | | |

**TableS3. Prediction accuracy for each cross using bi-allelic or parental model in each trait.**

|  | **Perillaldehyde** | | **Rosmarinic Acid** | | |
| --- | --- | --- | --- | --- | --- |
|  | **Bi-allelic model** | **Parental model** | **Bi-allelic model** | **Parental model** |  |
| **Crs1** | 0.501 | 0.533 | 0.062 | 0.039 |  |
| **Crs2** | 0.295 | 0.342 | 0.084 | 0.216 |  |
| **Crs3** | 0.187 | 0.264 | 0.088 | 0.154 |  |
| **Crs4** | 0.237 | 0.337 | 0.281 | 0.355 |  |
| **Crs5** | -0.067 | -0.093 | 0.14 | 0.108 |  |
| **Crs6** | 0.361 | 0.48 | 0.196 | 0.165 |  |
| **Crs7** | 0.208 | 0.218 | -0.012 | -0.041 |  |
| **Crs8** | 0.054 | 0.003 | 0.085 | -0.028 |  |

**Fig. S1.** Phenotypic distributions of perillaldehyde, rosmarinic acid, and anthocyanin in the F₃ and F₄ generations. Panels (a) and (b) show scatter plots of rosmarinic acid and perillaldehyde contents in the F₃ and F₄ generations, respectively. Panel (c) shows the distribution of anthocyanin index in the F₃ generation. Red, green, and blue points or bars represent the S827, S840, and S844 populations, respectively.

**Fig. S2.** Genomic prediction (GP) accuracy and genomic heritability for each trait in the F₃ and F₄ generations using ‘Sekiho’ as the reference genome. GP accuracy was evaluated using the BayesB model, whereas genomic heritability was estimated using the GBLUP model. Panels (a) and (b) show the results for the F₃ generation, and panels (c) and (d) show those for the F₄ generation. Panels (a) and (c) indicate GP accuracy, while panels (b) and (d) indicate genomic heritability. Red, green, and blue bars represent the S827, S840, and S844 populations, respectively. Traits measured in the F₃ generation were perillaldehyde, rosmarinic acid, and anthocyanin (three measurement dates), whereas in the F₄ generation, perillaldehyde and rosmarinic acid were measured.

**Fig. S3.** Schematic representation of the method used to infer allele origin in the G₂ generation. A denotes alleles derived from ‘Sekiho’, B₁ from ‘st27’, and B₂ from ‘st40’. Panel (a) illustrates cases where two F₄ individuals possessed B alleles of the same origin, whereas panel (b) shows cases where the two individuals carried B alleles of different origins. For each SNP genotyped in the F₄ generation, the combinations of genotypes between the two individuals constituting a cross are shown. The combination AA–AA is omitted because it necessarily results in an AA genotype in the G₂ generation. Patterns symmetric with respect to B₁ and B₂ are also not shown.

**Fig. S4.**Scatter plot of perillaldehyde and rosmarinic acid phenotypes in the F₄ generation. Two red lines indicate the phenotypic values of the cultivar ‘Sekiho’ measured in the same field and in the same year. Black circles represent the five F₄ individuals used for crosses selected based on predicted additive genotypic values of G₂ progeny, whereas black triangles indicate the two individuals used for crosses selected by phenotypic selection. The seven individuals used to generate the G₂ generation are labeled with their individual IDs and the names of their corresponding crosses.

**Fig. S5.** Prediction accuracy of perillaldehyde and rosmarinic acid in the G₂ generation using the bi-allelic and parental models. Colors indicate different cross pairs. The x- and y-axes represent observed and predicted values, respectively. Panels (a) and (b) show the prediction accuracy for perillaldehyde, and panels (c) and (d) show that for rosmarinic acid. Panels (a) and (c) correspond to the bi-allelic model, whereas panels (b) and (d) correspond to the parental model. The numbers in the lower right corners of each panel indicate the Pearson correlation coefficients between observed and predicted values.

**Fig. S6.** Linkage disequilibrium (LD) in the G₂ generation based on actual and simulated genotypic data. Panel (a) shows LD calculated from the actual genotypic data, and panel (b) shows LD calculated from the simulated genotypic data. LD was estimated as the correlation between pairs of markers. The red line represents the smoothed trend.
